# Reinforced Epoxy Composites Modified with Functionalized Graphene Oxide

**DOI:** 10.3390/polym14020338

**Published:** 2022-01-16

**Authors:** Anton Mostovoy, Andrey Shcherbakov, Andrey Yakovlev, Sergey Arzamastsev, Marina Lopukhova

**Affiliations:** 1 Laboratory “Modern Methods of Research of Functional Materials and Systems”, Yuri Gagarin State Technical University of Saratov, Polytechnichskaya St., 77, 410054 Saratov, Russia; 2 Laboratory “Support and Maintenance of the Educational Process”, Yuri Gagarin State Technical University of Saratov, Polytechnichskaya St., 77, 410054 Saratov, Russia; gassmed7@gmail.com; 3 Department “Chemistry and Chemical Technology of Materials”, Yuri Gagarin State Technical University of Saratov, Polytechnichskaya St., 77, 410054 Saratov, Russia; aw_71@mail.ru; 4 Department “Ecology and Technosphere Safety”, Yuri Gagarin State Technical University of Saratov, Polytechnichskaya St., 77, 410054 Saratov, Russia; bort740@mail.ru; 5 Department “Economics and Humanitarian Sciences”, Yuri Gagarin State Technical University of Saratov, Polytechnichskaya St., 77, 410054 Saratov, Russia; mlopuhova@yandex.ru

**Keywords:** epoxy-diane resin, graphene oxide, surface functionalization, homogenization, physical and mechanical properties

## Abstract

The possibility of using graphene oxide as a modifying additive for polymer fiber-reinforced composites based on epoxy resin and basalt roving has been studied. The content of graphene oxide in the system has been experimentally selected, which has the best effect on the physico-mechanical properties of the obtained polymer composite material. The efficiency of the modification of the graphene oxide surface with APTES finishing additives and aminoacetic acid, which provides chemical interaction at the polymer matrix–filler interface, has been considered. The influence of graphene oxide and functionalizing additives on the polymer curing process was investigated using the thermometric method and differential scanning calorimetry.

## 1. Introduction

Reinforced composites are widely used in a variety of industries such as construction, automotive, shipbuilding, aerospace, and more. They are appreciated for their unique properties, such as high strength, low weight, resistance to aggressive media, and the ability to quickly adjust the properties of polymer composite materials (PCM) in situations requiring a special approach [1,2].

Modern composites face increasingly stringent requirements in terms of physicochemical and mechanical characteristics, the improvement of which is impossible without understanding the influence of each component of the polymer system on the processes of structure formation and composite structure. The main problem in obtaining reinforced PCMs with high strength is the matrix, since it has much lower physical and mechanical characteristics than the reinforcing fiber. Thus, when operating at the ultimate load, the matrix is a weak link, microcracks appear in it, and the fiber peels off from the binder, which results in the polymer’s destruction [3]. In order to increase the strength characteristics of the matrix, various nanodispersed particles, such as carbon nanotubes [4,5]; particles of oxidized graphene [6,7,8]; graphene [9,10,11] and graphite [12]; silicon dioxide [13,14]; nanodiamonds [15,16]; potassium polytitanates [17]; metal nanoparticles [18]; titanium dioxide nanotubes [19]; and others, have widely been used recently. De Cicco et al. [20] reported that the modification of the epoxy matrix with various nanoparticles has been considered, as a result of which structure changes and the strength of the polymer matrix increases. The adhesion between the matrix and the fiber increases as well, which prevents the propagation of microcracks during deformation and provides the hardening of the epoxy composite. Obtaining high strength characteristics after adding nanoparticles depends on factors such as the following: optimal content in the system, absence of agglomerates, high adhesion of the filler, and chemical interaction with the matrix. The correct use of such modifiers requires a complete understanding of their effect on the processes of structure formation and the composite structure.

Currently, the most promising modification method for ensuring optimal strength in the system “matrix–filler” is the functionalization of the surface of a nanoscale filler. This method consists in grafting chemical groups capable of reacting with the matrix onto the surface of nanodispersed particles, forming stable chemical bonds [21]. At the same time, functionalization contributes not only to an increase in strength characteristics of the finished composite but also to a uniform distribution of nanoparticles during the homogenization process. At the moment, there are several main methods of functionalization, which include treatment with various carboxylic acids, silanes, amines, and physical treatment methods.

Ma et al. [22] indicated that in order to improve the interfacial interaction between the epoxy matrix and graphene oxide particles, treating the latter with cyanuric chloride and diethylenetriamine was proposed in order to form new functional groups on the surface. Ma et al. claimed that the addition of modified GO into the composite improves covalent bonds, mechanical adhesion, and wettability between carbon fiber and the matrix, which is proved by an increase in physical and mechanical characteristics.

Thus, the addition of functionalized GO in an amount of 1% of the binder mass into the epoxy composite reinforced with carbon fiber increased the interfacial (IFSS) and interlaminar (ILSS) shear strength, as well as the ultimate strength in bending by 104%, 100%, and 78%, respectively, as compared to the original composite. Ferreira et al. [23] studied the possibility of treating GO with hexamethylenediamine (HMDE), aiming to replace oxygen on the GO surface with HMDE groups. New functional groups made it possible to improve the interaction between the matrix and GO, as well as their distribution in the system, which made it possible to increase polymer hardness by 33% as compared to unmodified GO.

Nonahal et al. [24] studied the effect of functionalization of GO with aliphatic amines on curing an epoxy composite. By using DSC, they found that the untreated GO reduces heat effects (makes it difficult for the curing reaction to proceed) due to steric hindrance. At the same time, the addition of the functionalized GO with an increase in amine chain length improved chemical interaction with the epoxy resin, which was observed in a significant increase in heat release.

In our study, the modifier aminosilane APTES and aminoacetic acid containing groups capable of interacting with the functional groups of the polymer matrix and GO were selected as functionalizing additives [21].

Glass, basalt, and carbon fibers are most widely used as reinforcing fibers for the production of structural materials. Recently, basalt fiber has attracted increasing attention as a reinforcing filler for unidirectional PCMs. Modern basalt fiber has similar characteristic to a glass one and even surpasses it in some respects. Thus, composites based on basalt roving have a higher strength and modulus of elasticity in tension [25], which is ensured by their better adhesion to the polymer matrix and higher strength characteristics of the fiber itself. Due to their characteristics, basalt-based plastics currently occupy a leading place in the PCM market in terms of price–quality ratio, possessing a price comparable to that of the fiberglass. They are superior in strength characteristics and only slightly inferior in this indicator to carbon-fiber reinforced plastics, but they are much cheaper. This makes basalt plastics more attractive for commercial use, which results in the search for further methods for their modification.

Currently, there is a large number of works devoted to the effect of various modifying additives on the structure and physical and mechanical properties of composites based on epoxy resins reinforced with basalt fiber. Having analyzed these studies, we can draw a conclusion that improving the physico-mechanical properties of composites due to grafting various functional groups onto the surface of nanoparticles is an effective method that has not been properly studied yet [23,26].

The aim of this study is to study the effect of functionalized graphene oxide on physical and mechanical properties, structural features, and processes of structure formation of a unidirectional reinforced epoxy composite.

In this article, amino-functionalized graphene oxide was prepared by chemical modification of the surface of a graphene oxide by using γ-aminopropyltriethoxysilane (APTES) and aminoacetic acid and used to obtain hybrid reinforced epoxy composites. The results showed that the functionalization and chemical compatibility of APTES-treated and aminoacetic acid-treated graphene oxid with epoxy composition provides their best dispersion in the epoxy composition, which had important influences on curing behavior, structure, and physicochemical and mechanical properties of the fiber-reinforced epoxy composites. This study shows an important reference value for modification, optimization, and design of epoxy composites for the production of high performance graphene oxide/basalt fibers/epoxy hybrid nanocomposites.

## 2. Materials and Methods

### 2.1. Materials

As the objects of the study, we used cable-stayed stretchers based on ED-20 epoxy resin (CHIMEX Limited, St. Petersburg, Russia) reinforced with basalt roving (BR) (NRB13-1200-KB12, OOO Kamenny Vek, Dubna, Russia) and cold cured polyethylene polyamine (PEPA) (CHIMEX Limited, St. Petersburg, Russia). Electrochemically synthesized graphene oxide (GO) was used as a structuring additive [27].

The surface of the GO was functionalized with γ-aminopropyltriethoxysilane (APTES) manufactured by Penta-91 (Moscow, Russia) and aminoacetic acid (NH_2_CH_2_COOH) (Component-Reaktiv, Moscow, Russia). For this, 0.5 g of GO was dispersed in 100 mL of the H_2_O: APTES/NH_2_CH_2_COOH (95:5) solution for 10 min, using an ultrasonic homogenizer. Then, the suspension was boiled by using a reflux condenser at 80 °C for 12 h with constant low speed stirring at a speed of 100 rpm. When modified with APTES solution, the pH of the mixture was brought to 5 by means of acetic acid. The reasons for the choice of an acidic medium were an increase in the level of silanol formation and a decrease in self-condensation reactions between hydrolyzed silanol groups. The resulting suspension was centrifuged and washed twice with water in order to remove excess aminosilane around the GO particles. Then, the product was dried at 80 °C in the oven.

### 2.2. Preparation of Epoxy Composites

GO was added into the epoxy resin as a modifying additive in the amount of 0.05–0.5 parts by weight (Figure 1.1). In order to increase the uniformity of distribution and prevent the aggregation of GO particles, the ultrasonic treatment of the composition by an ultrasonic disperser UZDN-2T (UkrRosPribor, Sumy, Ukraine) was conducted, Figure 1.3. The ultrasonic exposure parameters were a frequency of 22 ± 2 kHz and a duration of 30 min (10 cycles of 3 min); a water cooling circuit was used to remove heat from the binder.

Reinforced composites were produced by using the following technology: Basalt fiber roving (BR) was impregnated with an epoxy composition mixed with a hardener (the ratio of epoxy resin ED-20, BR, and hardener PEPA was 100 parts by mass of ED-20, 300 parts by mass of BR, and 15 parts by mass of PEPA), Figure 1.5, and evenly wound on two bushings (Figure 1.6) possessing grooves on the outer surface. Then, one bushing rotated around an axis passing through the centers of the bushings and twisted the threads. The resulting bundle was stretched with a force of 100 N for the uniform tension of all areas and hardened during 24 h with a subsequent stepwise heat treatment at 90 ± 5 °C for 2 h at 120 ± 5 °C for 2 h.

**Figure 2:**
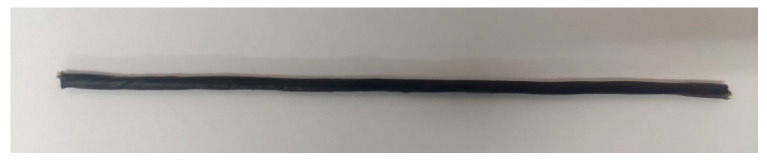
Photograph of the final product.

Photograph of the final product is shown in Figure 2.

### 2.3. Testing of the Composites

The determination of tensile strength and corresponding elastic module was carried out according to the ISO 178: 2010 standard, and tests were carried out at room temperature on a WDW-50E universal electromechanical testing machine from Time Group Inc. (Beijing, China) at a speed 5 mm/min; the tests were carried out on samples in the form of cylinders that are 4 mm in diameter and possessing a length of 150 mm. The determination of flexural strength and corresponding elastic module was carried out according to ISO 527-2:2012 standard, and tests were carried out at room temperature on a WDW-5E universal electromechanical testing machine from Time Group Inc. (Beijing, China) at a speed 10 mm/min; the tests were carried out on samples in the form of cylinders that are 4 mm in diameter and possessing a length of 80 mm. An LCT-50D pendulum pile driver (Beijing United Test Co., Ltd., Beijing, China) was used to determine impact strength according to ISO 179-1: 2010, and the tests were carried out on samples in the form of cylinders with 4 mm diameter and a length of 80 mm. FT-IR spectroscopy was carried out by using the Shimadzu IRTracer-100 (Tokyo, Japan). The study of surface morphology of the samples was carried out by using an Aspex EXplorer (Delmont, PA, USA) scanning electron microscope with a built-in energy-dispersive detector. The changes in mass, the rate of change in mass, and the magnitude of thermal effects during heating of the samples were studied by using the method of thermogravimetric analysis using a derivatograph MOM Q-1500 D Paulik-Paulik-Erdey (Budapest, Hungary) under experimental conditions: sample—100 mg; medium—air; heating interval—up to 1000 °C; heating rate—10 °C/min; and relative error did not exceed 1%. The self-heating temperature of the sample during the curing of the epoxy composition was determined according to the procedure described in [28]. In the DSC method, a Thermal Analyzer DTAS-1300 (Samara, Russia) was used with a sample weight of 20 mg and a heating interval up to 400 °C at a heating rate of 16 °C/min. The heat was determined through the heat flux—the derivative of heat over time. Heat fluxes were measured by the temperature difference at two points of the measuring system at the same time. The degree of curing of epoxy composites was determined by extracting samples of the crushed material with acetone in a Soxhlet apparatus. A sample of finely ground material weighing 1 g is poured with 20 mL of acetone and extracted in the apparatus; then, the material is dried, and the dry residue is weighed with an accuracy of 0.0001 g. The change in weight is calculated by the following formula: Δm = (m_1_ − m_2_)·100/m_1_, where m_1_—initial sample weight; and m_2_—weight of the sample after extraction and drying. The degree of curing (X) was determined by the following formula: X = 100 − Δm [28].

## 3. Results and Discussion

The key indicator of unidirectional reinforced composite materials is tensile breaking stress, as a result of which the selection of the amount of GO was carried out using this indicator as the dominant one. GO was added into the epoxy composition in the amount of 0.05–0.5 parts by mass. The optimal amount that provides the best physical and mechanical characteristics is 0.1 part by mass, Table 1, which increases the modulus of elasticity and tensile strength by 22–25% as compared to the unfilled composite. It was found that the positive effect of the addition of GO in various concentrations on the elastic modulus fluctuated within the experimental error.

In order to reduce the tendency of nanomaterials to aggregate and increase their adhesive ability, it is necessary to functionalize them and develop effective methods for the physical modification of epoxy compositions that will provide effective adhesive interaction at the polymer matrix/nanoscale filler interface. One of the promising methods of functionalization is the surface treatment of fillers with compounds that provide chemical interaction between the filler and the polymer matrix and reduce the polydispersity of the filler, which will enhance the physical and mechanical properties of composites based on them [23,26].

**Figure 3:**
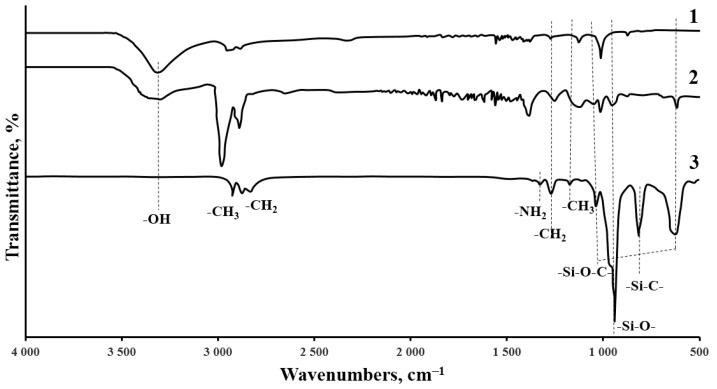
FT-IR spectroscopy of samples: 1—initial GO; 2—GO, after APTES treatment; 3—APTES.

In the current study, APTES and aminoacetic acid are used as such compounds. They contain amino groups capable of interacting with epoxy groups of the oligomer, as well as silanol and carboxyl groups that will provide interaction with the nanofiller. The formation of strong bonds between GO and APTES was proved by FT-IR spectroscopy (Figure 3). As observed from the sample spectrum after modification, the vibration intensity of hydroxyl groups (3400 cm^−1^), which are involved in the formation of the APTES layer, significantly decreases. Moreover, vibration peaks corresponding to APTES appear in the GO_APTES_ spectrum; a broad maximum is also observed at about 1100 cm^−1^, which indicates the presence of Si-O bonds in the sample.

The chemical interaction of the functional groups of APTES and the epoxy oligomer was proved by us previously [5,17,21].

**Figure 4:**
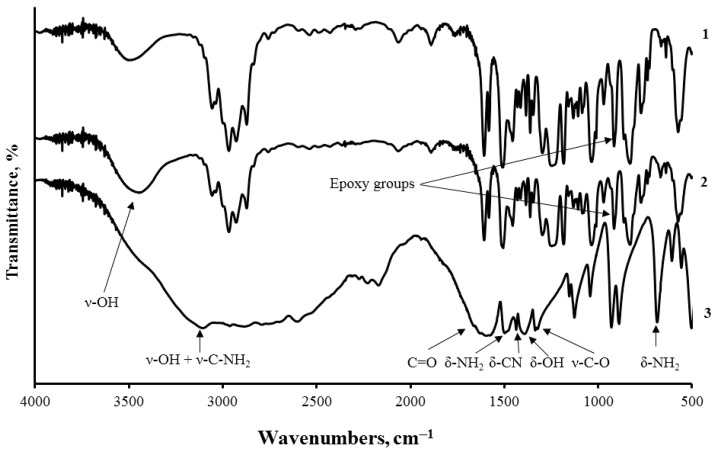
FT-IR spectroscopy of samples: 1—ED-20; 2—ED-20 + aminoacetic acid; 3—aminoacetic acid.

Aminoacetic acid contains an amine group that can interact with the epoxy ring of the resin when added into the composite. After mixing aminoacetic acid and ED-20 without PEPA, a significant (by 38%) decrease in intensity of the absorption peak corresponding to the epoxy ring (910 cm^−1^) was observed. The vibration peak of hydroxyl groups (at 3470 cm^−1^) formed during the opening of the epoxy cycle increases (by 22%), which proves the chemical interaction between the components (Figure 4).

**Figure 5:**
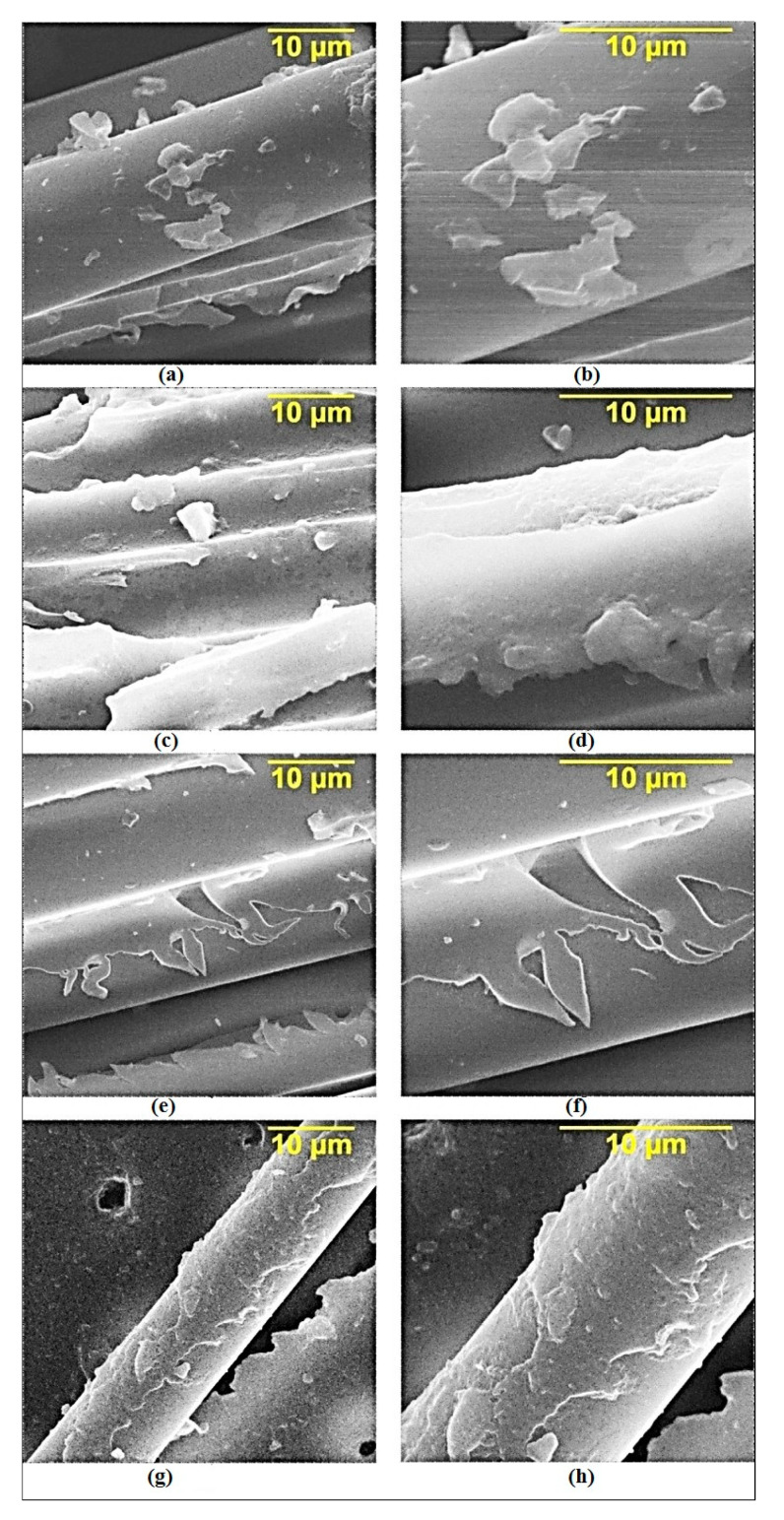
SEM data of unidirectional reinforced epoxy composites: (**a**,**b**)—original composite; (**c**,**d**)—composite with 0.1 part by mass of GO; (**e**,**f**)—composite with 0.1 part by mass of GO functionalized with APTES; (**g**,**h**)—composite with 0.1 part by mass of GO functionalized with aminoacetic acid.

An accurate understanding of the effect of the filler and the modifying agents is impossible without studying the sample’s fractography, which was studied by using the SEM images of the composites. The fracture surface of the samples shows the effect of various modifying additives on the structure of the resulting composite. Figure 5a,b clearly show that the unmodified epoxy resin has poor adhesion to the fiber, which is observed from the small amounts of epoxy binders on the fiber’s surface. The addition of GO into the composition of the composition results in an increase in the adhesion of the matrix to the fiber and an increase in its strength, which is observed in an increase in the matrix amount on the fiber’s surface (Figure 5c,d).

The grafting of APTES and aminoacetic acid onto the surface of the GO ensures the cohesive nature of the composite’s destruction. This conclusion can be drawn by paying attention to the thin layer of the matrix remaining on the surface of the fibers (Figure 5e–h). The cohesive nature of the destruction made it possible to obtain high physical and mechanical characteristics.

**Figure 6:**
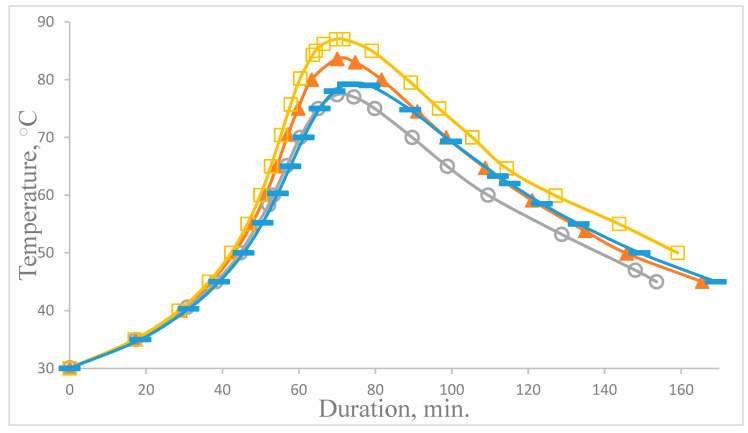
Kinetic curves of epoxy composite curing: ▲—ED-20 + BR + PEPA; ○—ED-20 + BR + PEPA + GO; □—ED-20 + BR + PEPA + GO_(APTES);_ ▬—ED-20 + BR + PEPA + GO_(aminoacetic acid)_.

It is impossible to completely understand the effect of GO on cross-linked polymers without studying the curing processes of composites in the presence of a filler and modifying additives capable of affecting the kinetics of the polymerization reaction [28,29,30]. Analysis of the kinetic curves of curing shows that the addition of GO and the subsequent functionalization of the GO surface with chemically active substances affects the processes of the structure formation of the epoxy composite during curing (Figure 6).

**Figure 7:**
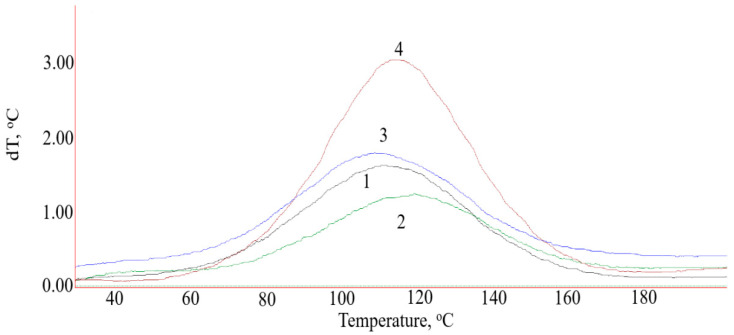
DSC data of epoxy composite: 1—original composite; 2—composite with 0.1 part by mass of GO; 3—composite with 0.1 part by mass of GO functionalized with aminoacetic acid; 4—composite with 0.1 part by mass of GO functionalized with APTES.

The addition of GO into the epoxy composition slows down the curing process, which is confirmed by an increase in the duration of the gelation process from 37 to 41 min and the curing process from 70 to 76 min, while the maximum curing temperature decreases from 84 to 77 °C (Table 2). The data from differential scanning calorimetry confirm the results obtained (Figure 7), and a decrease in reaction enthalpy and an increase in temperature of the onset of curing from 53 to 60 degrees are observed (Table 3).

The addition of GO finished with APTES and aminoacetic acid into the epoxy composition initiates a polymerization reaction, which is proved by a reduction in the duration of the gelation process from 41 to 32 min and a decrease in temperature at the start of curing from 60 to 51–56 °C (Table 2 and Table 3), with an increase in the maximum curing temperature from 77 to 80–87 °C and in the enthalpy of reaction, which indicates that the functional groups of APTES and aminoacetic acid react with the functional groups of the epoxy oligomer. In addition, it was observed that the introduction of electrochemically synthesized graphene oxide, both initial and functionalized, in contrast to graphene oxide synthesized by the Hammers method [31,32] increases the glass transition temperature of the epoxy composite from 77 to 85–91 °C (Table 3). This effect is probably associated with a specific functionalization of GO particles. During the electrochemical oxidation of graphite, a GO structure is formed with a high oxygen concentration in the composition of functional groups both at the periphery of the GO particle and in the interlayer spaces, due to the fact that the electrochemical oxidation of graphite is accompanied by the release of oxygen, which results in the formation of over-oxidized compounds [33].

Thermogravimetric analysis showed an increase in thermal stability of the composite in the temperature range of 200–350 °C with the addition of GO finished with APTES and aminoacetic acid (Table 4).

The organization of chemical interactions at the filler (GO)–binder interface due to the treatment of the filler surface with APTES/aminoacetic acid results in the improvement of physical and mechanical properties of epoxy composites: The tensile strength increases by 16% and 10%; the elastic moduli increases by 31% and 19%; the ultimate strength in bending increases by 9% and 13%; and the changes in the elastic modulus are within experimental error, while impact strength decreases by 18% (Table 5). An increase in the physical and mechanical characteristics upon the introduction of functionalized GO is due to the fact that strong chemical bonds are formed at the polymer matrix–filler interface, which requires additional energy for their destruction; in addition, as shown by the study of the fracture structure of composites, the grafting of APTES and aminoacetic acid onto the surface of GO ensures the cohesive nature of the composite’s destruction.

Developed composites have physico-mechanical characteristics comparable to those of existing analogs and often cases exceeding them (Table 6).

## 4. Conclusions

Modification of the epoxy composition affects the physical and mechanical properties, processes of the structure formation, morphology, and thermal stability of reinforced epoxy composites. The functionalization of the GO surface with APTES additives and aminoacetic acid providing a chemical interaction at the polymer matrix–filler interface and preventing aggregation of GO particles results in the strengthening of epoxy composites.

Grafting APTES and aminoacetic acid onto the GO surface ensures the cohesive nature of the destruction of the composite, which makes obtaining enhanced physical and mechanical characteristics possible.

It has been established that the addition of GO and the subsequent functionalization of the GO surface with chemically active substances affects the processes of structure formation of the epoxy composite during curing. The addition of GO finished with APTES and aminoacetic acid into the epoxy composition initiates the polymerization reaction, which is confirmed by a decrease in the duration of the gelation process and a decrease in the temperature of the start of curing. At the same time an increase in the maximum curing temperature and enthalpy of reaction is noted, which indicates that the functional groups of APTES and aminoacetic acid react with the functional groups of the epoxy oligomer. Moreover, an increase in the glass transition temperature of the epoxy composite is noted.

The use of functionalized GO made it possible to increase the thermal stability of the composite in the temperature range of 200–350 °C.

## Figures and Tables

**Figure 1:**
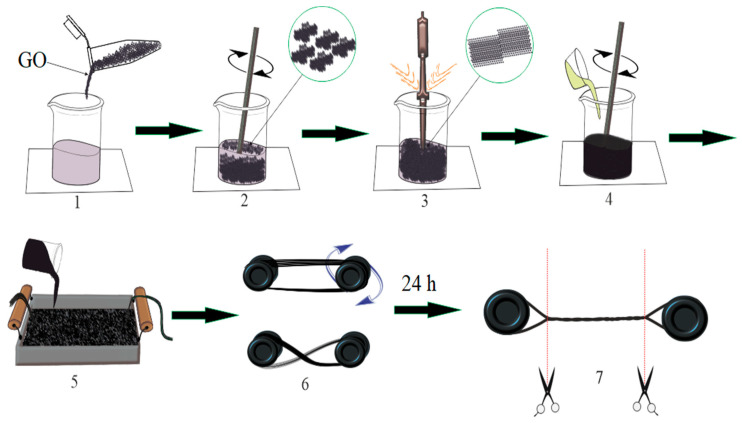
Scheme of obtaining a reinforced composite.

**Table 1:** Properties of reinforced epoxy composites.

Composition, Parts by Mass	Tensile Strength, MPa	Tensile Modulus, GPa
100ED-20 + 300BR + 15PEPA	1460 ± 60	76 ± 3.0
100ED-20 + 300BR + 15PEPA + 0.05GO	1690 ± 65	92 ± 3.5
100ED-20 + 300BR + 15PEPA + 0.075GO	1670 ± 62	93 ± 3.6
100ED-20 + 300BR + 15PEPA + 0.1GO	1830 ± 70	86 ± 3.3
100ED-20 + 300BR + 15PEPA + 0.5GO	1600 ± 60	85 ± 3.2

Note: ±—standard deviation.

**Table 2:** Values of indicators of epoxy composition curing.

Composition, Parts by Mass	Duration of Gelation, min	Duration of Curing, min	Maximum Curing Temperature, °C	Curing Degree, %
100ED-20 + 300BR + 15PEPA	37	70	84	98.67
100ED-20 + 300BR + 15PEPA + 0.1GO	41	76	77	98.57
100ED-20 + 300BR + 15PEPA + 0.1GO_APTES_	32	69	87	98.39
100ED-20 + 300BR + 15PEPA + 0.1GO_aminoacetic acid_	35	73	80	98.73

**Table 3:** Values of curing indices by differential scanning calorimetry.

Composition, Parts by Mass	T_start_–T_end_ T_max_, °C	T_glass_, °C	H, J/g
100ED-20 + 300BR + 15PEPA	53–179 108	77	277
100ED-20 + 300BR + 15PEPA + 0.1GO	60–165 113	85	190
100ED-20 + 300BR + 15PEPA + 0.1GO_APTES_	51–173 113	91	490
100ED-20 + 300BR + 15PEPA + 0.1GO_aminoacetic acid_	56–169 111	87	305

Note: T_start_, T_end_—temperature of the start and end of the curing process; T_max_—the temperature of the maximum heat release during curing; H—thermal effect of reaction.

**Table 4:** Thermogravimetric analysis results.

Composition, Parts by Mass	Weight Loss of Epoxy Matrix, %, at Thermolysis Temperatures, °C								
	200	250	300	350	400	450	500	550	600
100ED-20 + 300BR + 15PEPA	0	0.1	12.9	26.4	38.9	53.7	74.0	94.7	100
100ED-20 + 300BR + 15PEPA + 0.1GO	0	1.5	14.3	27.2	40.1	55.7	75.4	94.6	100
100ED-20 + 300BR + 15PEPA + 0.1GO_APTES_	0	0	14.5	32.9	47.9	65.1	87.3	94.5	100
100ED-20 + 300BR + 15PEPA + 0.1GO_aminoacetic acid_	0	0	11.0	24.6	38.6	56.6	79.0	96.9	100

**Table 5:** Properties of reinforced epoxy composites.

Composition, Parts by Mass	σ_ben,_ MPa	E_ben_, GPa	σ_ten_, MPa	E_ten_, GPa	a_im_, kJ/m^2^
100ED-20 + 300BR + 15PEPA	600 ± 25	26 ± 1.0	1460 ± 60	76 ± 3.0	300 ± 10
100ED-20 + 300BR + 15PEPA + 0.1GO	600 ± 25	24 ± 0.8	1830 ± 70	85 ± 3.3	290 ± 8
100ED-20 + 300BR + 15PEPA + 0.1GO_APTES_	650 ± 26	24 ± 0.8	2130 ± 82	110 ± 4.2	240 ± 8
100ED-20 + 300BR + 15PEPA + 0.1GO_aminoacetic acid_	690 ± 28	24 ± 0.8	2020 ± 80	100 ± 4.1	250 ± 8

Note: σ_ben_—bending stress; E_ben_—modulus of elasticity in bending; σ_ten_—tensile strength; E_te_—tensile modulus of elasticity; a_im_—impact strength.

**Table 6:** Comparison of developed composites with analogues.

Composition, Parts by Mass	σ_ben,_ MPa	E_ben_, GPa	σ_ten_, MPa	E_ten_, GPa	a_im_, kJ/m^2^
100ED-20 + 300BR + 15PEPA + 0.1GO_APTES_	650 ± 26	24 ± 0.8	2130 ± 82	110 ± 4.2	240 ± 8
100ED-20 + 300BR + 15PEPA + 0.1GO_aminoacetic acid_	690 ± 28	24 ± 0.8	2020 ± 80	100 ± 4.1	250 ± 8
Analogs					
Epoxy resin + Glass fiber + Hardener SX10M [34]	224 ± 13	15 ± 1.4	582 ± 12	16 ± 0.5	-
Epoxy resin + Basalt fiber + Hardener SX10M [34]	505 ± 23	23 ± 1.1	506 ± 21	25 ± 1.1	-
Epoxy resin + UD carbon fiber + amine hardener + 0.5 wt% CNT [4]	-	-	1723.5 ± 46	134 ± 2.4	-
Epoxy resin + woven carbon + nanoparticles 3 wt % Al_2_O_3_ + hardener [35]	440.6 ± 20	-	759 ± 23	-	-
Epoxy resin + carbon fiber + CNT-PA6 [36]	1002 ± 50	58.3 ± 1.8	-	-	75 ± 3

Note: σ_ben_—bending stress; E_ben_—modulus of elasticity in bending; σ_ten_—tensile strength; E_ten_—tensile modulus of elasticity; a_im_—impact strength.

## Data Availability

The data presented in this study are available on request from the corresponding author.
